# Definitely maybe: can unconscious processes perform the same functions as conscious processes?

**DOI:** 10.3389/fpsyg.2015.00584

**Published:** 2015-05-06

**Authors:** Guido Hesselmann, Pieter Moors

**Affiliations:** ^1^Visual Perception Laboratory, Department of Psychiatry and Psychotherapy, Charité-Universitätsmedizin Berlin, Berlin, Germany; ^2^Laboratory of Experimental Psychology, University of Leuven (KU Leuven), Leuven, Belgium

**Keywords:** consciousness, social psychology, cognitive psychology, visual perception, continuous flash suppression

## Abstract

Hassin recently proposed the “Yes It Can” (YIC) principle to describe the division of labor between conscious and unconscious processes in human cognition. According to this principle, unconscious processes can carry out every fundamental high-level cognitive function that conscious processes can perform. In our commentary, we argue that the author presents an overly idealized review of the literature in support of the YIC principle. Furthermore, we point out that the dissimilar trends observed in social and cognitive psychology, with respect to published evidence of strong unconscious effects, can better be explained by the way how awareness is defined and measured in both research fields. Finally, we show that the experimental paradigm chosen by Hassin to rule out remaining objections against the YIC principle is unsuited to verify the new default notion that all high-level cognitive functions can unfold unconsciously.

## The “Yes It Can” Principle

In *Perspectives on Psychological Science*, Hassin has recently proposed a novel principle, the “Yes It Can” (YIC) principle, to describe the division of labor between conscious and unconscious processes in human cognition (Hassin, 2013). In brief, the YIC principle states that “unconscious processes can carry out every fundamental high-level [cognitive] function that conscious processes can perform” (p. 195). According to Hassin, two observations lend *a priori* plausibility to the YIC principle. First, conscious processing has been shown to be severely capacity-limited (Baddeley, 2007). Second, some theories suggest that conscious awareness as we experience it today might be a relatively recent evolutionary development (Dennett, 1991). Hassin concludes that, therefore, “fundamental cognitive functions are likely to occur outside of conscious awareness” (p. 195).

The author then reviews a wide and diverse range of supporting evidence from cognitive and social psychology, as well as the cognitive neurosciences. The selected studies unanimously show that high-level cognitive functions that were previously thought of as requiring consciousness can indeed occur non-consciously, i.e., without awareness of the relevant stimuli or without awareness of the influence of the relevant stimuli, respectively. In particular, he presents empirical data from four different subsets of cognitive functions, namely cognitive control (e.g., conflict adaptation), goal pursuit (e.g., goal priming), information broadcasting (e.g., semantic priming), and reasoning (e.g., decision making).

What is more, Hassin provides a surprisingly simple recipe for testing whether a cognitive operation can be performed unconsciously. The operation should be stripped “into its basic-level functions” and submitted to an experimental design which “(a) tests the functions while (b) allowing the processes to occur non-consciously” (p. 200). The YIC principle predicts that based on this recipe any cognitive function of interest will be found to be performed unconsciously. Hassin concludes his paper by acknowledging that default notions are of great importance for the advancement of science, and he expresses his hope that the YIC principle will help establish a new default mode of thinking about the abilities of the unconscious.

## Hassin’s Idealized Review of the Literature

In support of the YIC principle, Hassin presents an idealized review of the literature, without a single reference to conflicting evidence, methodological debates or recent discussions of the reproducibility of psychological studies. This selective focus inevitably creates the impression that indeed the majority of cognitive functions have already been proven to function unconsciously. For example, one major line of research reviewed by Hassin suggests that complex decisions might benefit from a period of time in which participants engage in what is referred to as “unconscious thought” (Dijksterhuis et al., 2006). What remains unmentioned is that the theory’s main claims and decision task have been met with severe methodological as well as theoretical criticism (Gonzalez-Vallejo et al., 2008; Waroquier et al., 2009), and that further empirical tests of the unconscious thought theory’s predictions yielded no evidence in favor of these predictions (Huizenga et al., 2012); for a recent meta-analysis and large-scale replication attempt of the unconscious thought advantage, see (Nieuwenstein et al., 2015).

In his review of the literature, Hassin also cites his own work on priming which suggests that both invisible (Hassin et al., 2007) and visible yet subtle (Carter et al., 2011) exposure to national flags significantly changed political attitudes and voting intentions up to 8 months later. Only after publication of the YIC principle, a large-scale, preregistered “many labs” replication project did not replicate the finding that visible yet subtle exposure to the American flag increased conservatism among US participants (Klein et al., 2014). While replication should become a standard tool in psychological science to confirm the accuracy of empirical findings, clarify the conditions under which an effect can be observed, and estimate the true effect size (Open Science Collaboration, 2012; Simons, 2014), failures to replicate often leave relevant questions unanswered, in particular questions about the specificity of the underlying theories (Klein, 2014)^1^. It is important to note that Hassin and colleagues supported the replication attempt and acknowledged the failure to replicate the flag priming effect, but also raised the issue of whether it was a “conceptual” rather than “direct” replication, and they addressed the importance of identifying potential moderators of the original phenomenon and of understanding the theory behind the effects (Ferguson et al., 2014).

In sum, we believe that a nuanced review of the literature on unconscious processing would not only be more adequate, but also convey a more veridical picture telling us “definitely maybe” rather than “yes it can,” as an answer to the question of whether unconscious processes perform the same functions as conscious processes.

## Unconscious Priming in Social and Cognitive Psychology

In a synthetic attempt, Hassin brings together two largely separate research traditions in his overview of the literature, namely the research on unconscious priming effects in social psychology, on the one hand, and cognitive psychology, on the other. As pointed out by Hassin, the scope and limits of semantic subliminal priming have been debated among cognitive psychologists over the course of decades (Eriksen, 1960; Marcel, 1983; Greenwald, 1992; Kouider and Dehaene, 2007), with views shifting almost pendulum-like across time, while research in the field of social psychology, almost simultaneously, accumulated evidence for unconscious priming effects following a monotonically upward trend, in particular for behavioral priming effects. For example, conceptual replications of the influential study showing that subtle primes could affect overt behavior (Bargh et al., 1996) are abundant. Amongst others, participants primed with the concept of “politician” wrote essays that were considerably longer than did control participants (Dijksterhuis and Van Knippenberg, 2000), and the presence of a backpack in the experimental room primed more cooperative behavior, while the presence of a briefcase primed more competitive behavior (Kay et al., 2004).

In a recent opinion paper published in *Perspectives on Psychological Science* (as part of a special section described in footnote 1), Dijksterhuis estimated that there are between 200 and 400 empirical behavioral priming papers by now (Dijksterhuis, 2014). To explain the dissimilar trends in social and cognitive psychology, Hassin argues that “the unconscious is likely to engage in motivationally relevant and interesting issues (such as goals, stereotypes, and incentives) more than in motivationally irrelevant and less interesting issues (such as the relations between chairs and tables)” (p. 201). As an alternative to this motivational account, Doyen et al. (2014) have convincingly elaborated three sources of conflict between the two research fields: awareness, processes, and replicability. With respect to awareness, the authors argue that in social psychology the absence of awareness is often assumed rather than tested, and when tests are conducted, they are below the standards widely used in cognitive psychology. Similarly, it has been suggested that in social psychology experiments on behavioral priming there has been a problematic shift from defining unconscious as “without awareness of the stimuli” (as in cognitive psychology) to “without awareness of the influence of the stimuli” (Stafford, 2014). Thus, rather than a new default mode of thinking about the abilities of the unconscious, the joint efforts of social and cognitive psychology should help establish a new default of measuring stimulus awareness in the diverse range of priming experiments. This stringent default should entail that for each experiment it first has to be defined which aspect of awareness matters and how it can be measured optimally (Doyen et al., 2014). From a statistical perspective, much progress has recently been made in the application of Bayesian statistics to the central problem in consciousness research of stating evidence for the null hypothesis, which is the case, for example, when the aim is to establish chance-level performance as a proof of objective unawareness of a stimulus (Dienes, 2015). Therefore, any new default in consciousness research should also involve well-informed thinking about statistical tests and their implications.

## Is Interocular Suppression Suited to Rule out Remaining Objections?

As Hassin argues in the paragraph *A Walk Through a Garden of Objections*, the YIC principle is often quickly criticized by devising examples of cognitive functions that would, almost by definition, require consciousness to fully unfold. Thus, a particularly strong test of the YIC principle would be to acquire evidence for the unconscious to be able to perform seemingly high-level cognitive functions such as reading and effortful arithmetic. This was exactly the goal of one experimental study which involved Hassin as senior author (Sklar et al., 2012). To assess the feasibility of unconscious reading and arithmetic, the authors used continuous flash suppression (CFS) to unconsciously present multiple-word verbal expressions or single-digit equations to observers. CFS refers to an interocular suppression technique in which discrepant images are presented to the different eyes at corresponding retinal locations (Tsuchiya and Koch, 2005; Yang and Blake, 2012). In consciousness research, there has always been a trade-off between different paradigms to present stimuli unconsciously to observers (Kim and Blake, 2005; Bachmann et al., 2007). For example, visual masking provides a reliable tool to present a stimulus in the absence of awareness, yet it suffers from a very short presentation time of this stimulus. Binocular rivalry provides a longer window to present stimuli unconsciously, but it is much harder to control how long a stimulus will be suppressed. In contrast, CFS provides the potential to present visual stimuli unconsciously from trial onset and for extended periods (i.e., on a second rather than millisecond timescale). This has rendered CFS an increasingly attractive technique to study the limits of unconscious processing^2^. As Sklar et al. (2012) argue, CFS is a “cutting edge masking technique” that probably will act as a “game changer” (p. 19614) in consciousness research due to its seemingly unlimited potential to present stimuli unconsciously for extended periods of time.

In their study, Sklar et al. (2012) use CFS in two different ways to study unconscious reading and arithmetic. With respect to reading, the semantic coherence and the affective value of multiple-word verbal expressions was manipulated, and the time it took for these expressions to break suppression was measured (hence “b-CFS” paradigm). The reasoning behind b-CFS is that differential suppression times for differential stimuli must be due to differences in processing during suppression if control measures can convincingly show that the stimuli show no intrinsic difference in detectability when no interocular suppression is involved. In these b-CFS experiments, Sklar et al. (2012) showed that semantically incoherent expressions broke suppression faster than semantically coherent ones and that the affective value of verbal expressions modulated suppression time such that increasing negativity of the expression lowered suppression times significantly. With respect to arithmetic, Sklar et al. (2012) presented single-digit equations with three terms but without result (e.g., “9–3–4=”) and tested whether these would influence the enumeration of a visible target number. Here, a significant congruency effect was observed for subtraction primes, but not for addition primes. For addition primes, the authors observed congruency effects only when single-digit equations with two terms were unconsciously presented and participants had to report whether a subsequently presented visible addition equation with two terms and result was correct or not. Based on their results, the authors concluded that the meaning of verbal expressions can be extracted unconsciously and that effortful arithmetic equations can be solved without awareness.

The study by Sklar et al. (2012) is not the only one that has used CFS and concluded that unconscious high-level (visual) processing is possible. Indeed, to name a few, studies have shown that word meaning (Costello et al., 2009) and word valence (Yang and Yeh, 2011) can be processed unconsciously, that scene congruency information can be extracted in the absence of visual awareness (Mudrik et al., 2011), that emotional information is processed during suppression (Yang et al., 2007) or that sexual orientation can bias the processing of unconsciously presented nude pictures (Jiang et al., 2006).

We would like to argue, however, that this enthusiasm for the potential of CFS to explore uncharted territories for consciousness research is most likely premature and farfetched. The principal reason for this argument is that all studies that embark on finding high-level unconscious processing during CFS ignore the representation of the stimulus while it is being suppressed. That is, any paradigm that renders a visual stimulus does so by interfering with the processing of the unconsciously presented stimulus in some way (Fogelson et al., 2014). If not, the stimulus would always be visible to the observer. CFS is closely related to binocular rivalry, a well-known interocular suppression technique, the mechanisms of which have been extensively investigated in the last decades (Blake and Logothetis, 2002; Sterzer, 2013). Given the rather limited cognitive processing during binocular suppression (Zimba and Blake, 1983; Blake, 1988; Cave et al., 1998; Kang et al., 2011), the default stance should thus be *not* to expect much high-level unconscious processing during CFS (Breitmeyer, 2014). Recent neuroimaging data suggests that the presence of CFS masks dramatically reduces neural activity related to the suppressed stimulus already in early visual cortex (Yuval-Greenberg and Heeger, 2013). Thus, the representation of the suppressed stimulus is expected to be rather limited to a loose collection of elemental features that are presumably coded in these early visual areas, despite the fact that the stimulus is presented unbeknownst to the observer for extended periods of time (Gayet et al., 2014). Indeed, considering the CFS literature as a whole sketches a more complicated and fuzzy picture about the extent to which high-level unconscious visual processing is possible under CFS (Heyman and Moors, 2014; Sterzer et al., 2014; Yang et al., 2014; Ludwig and Hesselmann, 2015). The interested reader is further referred to Dubois and Faivre (2014) for a special issue in *Frontiers in Psychology (Consciousness Research)* on the depth of unconscious processing as inferred from different suppression techniques. Similar to the picture sketched based on the behavioral priming literature, the picture emerging from the accumulating literature on unconscious processing during CFS is rather “definitely maybe” than “yes it can.”

## Concluding Remarks

In our commentary, we have presented arguments against the YIC principle which states that unconscious processes can carry out every fundamental high-level cognitive function that conscious processes can perform. We argued that the apparent strength of the YIC principle is based on an idealized review of the available literature and that it ignores the ongoing and lively methodological discussions on how to appropriately study unconscious processing. Furthermore, the proposed potential of a paradigm such as CFS to rule out any remaining objections regarding YIC appears to be overvalued and based on a similarly idealized reading of the literature. While we agree with Hassin that progress in science requires new ideas and defaults, we would argue that rather than “yes it can” a more skeptical “definitely maybe” is in much better accordance with the current state of affairs.

### Conflict of Interest Statement

The authors declare that the research was conducted in the absence of any commercial or financial relationships that could be construed as a potential conflict of interest.

## Footnotes

1 We would like to direct the reader’s attention toward two related special issues which appeared in Perspectives on Psychological Science: the November 2012 issue dedicated to the topics of replicability and research practices, and the January 2014 issue which features a special section focused on behavioral priming research and attemps at replication. A further target article on the replicability in psychology and open peer commentary can be found in a recent edition of the European Journal of Personality (Asendorpf et al., 2013).

2 For a complete CFS reference list, see http://www.gestaltrevision.be/en/resources/reference-guides/83-resources/reference-guides/343
